# 2025: Institutional and community involvement establishing ARresearch.org and innovative recruitment results in diverse registrants

**DOI:** 10.1017/cts.2017.240

**Published:** 2018-05-10

**Authors:** Jean McSweeney, David Robinson, Anthony McGuire, Pamela Christie, Sandra Hatley, Martha Rojo, Laura James

## Abstract

OBJECTIVES/SPECIFIC AIMS: To establish a state-wide research registry of diverse participants. METHODS/STUDY POPULATION: We garnered broad institutional and community support by involving TRI’s Community Engagement team, its Community Advisory Board (CAB), and 3 UAMS patient CABs in selecting Web site content, images, and colors. Using this feedback, the TRI Recruitment Unit (RU), in conjunction with UAMS Communications and the Center for Health Literacy, developed the materials and crafted comprehensive communication and recruitment strategies. The UAMS Center for Pacific Islander Health, Hispanic faculty, and CAB members translated materials. UAMS IT programmed the user-friendly site to allow registration from smartphones and i-Pads and linked to UAMS patient electronic health messages. RESULTS/ANTICIPATED RESULTS: The RU committee implemented successful innovative strategies, including recruiting at the Arkansas State Fair and ballgames, attended by people of all races, ages, and socio-economic levels. Using i-Pads at the sites, recruitment took <5 minutes/registrant. Within 8 months, >2400 participants from across Arkansas had joined the registry: 14% African-Americans, 8% Pacific Islanders, 5% Hispanic, and 3% Native American. DISCUSSION/SIGNIFICANCE OF IMPACT: Involving CAB multidisciplinary input to design and implement recruitment materials was highly successful. Despite challenges of recruiting under-represented groups, the registry includes 30% minorities. By tracking registrants’ demographics with Lime Survey software, the RU will prioritize future recruitment events to maximize diversity of registrants.

